# ‘Seed + expand’: a general methodology for detecting publication oeuvres of individual researchers

**DOI:** 10.1007/s11192-014-1256-0

**Published:** 2014-03-05

**Authors:** Linda Reijnhoudt, Rodrigo Costas, Ed Noyons, Katy Börner, Andrea Scharnhorst

**Affiliations:** 1 DANS, Royal Netherlands Academy of Arts and Sciences (KNAW), The Hague, The Netherlands; 2 Center for Science and Technology Studies (CWTS)-Leiden University, Leiden, The Netherlands; 3 Cyberinfrastructure for Network Science Center, School of Informatics and Computing, Indiana University, Bloomington, IN USA

**Keywords:** Author disambiguation, Publication oeuvre, Scalable methods

## Abstract

The study of science at the individual scholar level requires the disambiguation of author names. The creation of author’s publication oeuvres involves matching the list of unique author names to names used in publication databases. Despite recent progress in the development of unique author identifiers, e.g., ORCID, VIVO, or DAI, author disambiguation remains a key problem when it comes to large-scale bibliometric analysis using data from multiple databases. This study introduces and tests a new methodology called seed + expand for semi-automatic bibliographic data collection for a given set of individual authors. Specifically, we identify the oeuvre of a set of Dutch full professors during the period 1980–2011. In particular, we combine author records from a Dutch National Research Information System (NARCIS) with publication records from the Web of Science. Starting with an initial list of 8,378 names, we identify ‘seed publications’ for each author using five different approaches. Subsequently, we ‘expand’ the set of publications in three different approaches. The different approaches are compared and resulting oeuvres are evaluated on precision and recall using a ‘gold standard’ dataset of authors for which verified publications in the period 2001–2010 are available.

## Introduction

Creating correct linkages between a unique scholar authoring a work and her or his (possibly many) author name(s) is complex and unresolved. Authors might use anonymous and alias author names, names might be misspelled or change over time, e.g., due to marriage, and multiple scholars might have the very same name.^1^ Yet, science is driven by scholars, and the identification and attribution of works to individual scholars is important for understanding the emergence of new ideas, to measure the creative human capital of institutions and nations, to model the relationships and networks of researchers, and to forecast new scientific fields (Scharnhorst et al. 2012). With the ‘return of the author’ in bibliometrics (Scharnhorst and Garfield 2011); bibliometric indicators on the individual level (Hirsch 2005; Costas et al. 2010; Lariviere 2010; Vieira and Gomes 2011); and institutional evaluation based on the individual publication output of authors over longer time periods (van Leeuwen 2007; Zuccala et al. 2010), the ambiguity problems in allocating publications to authors have become more pressing (Costas and Bordons 2005; Costas et al. 2010). Different approaches to data collection at the individual level have been proposed in the literature (see the review by Smalheiser and Torvik 2009), although in many cases these approaches focus on the disambiguation of author in one single database (e.g. PubMED). Recently, systems of unique author identifiers offer a practical solution. Collaborative web-based information systems as ResearchGate, Mendeley, arXiv and Google Scholar require individual registration. Slowly one can observe a process of standardization with initiatives like ORCID which is matching different profiles like the Scopus Author Identifier and ResearcherID. However, they are not yet fully standardized and often rely on authors to register their own bibliographic profiles. Thus, the problem of automatically linking author names across publication, patent, or funding databases persists.

In this paper, we present a general methodology that combines information from different data sources^2^ to retrieve scientific publications covered in the Web of Science (WoS) during the period 1980–2011 for a given list of authors. Specifically, we trace the publications for 8,378 professors affiliated with at least one of the Dutch universities, as included in the *Nederlandse Onderzoek Databank* (NOD, Dutch Research Database) and displayed in the web portal NARCIS (National Academic Research and Collaborations Information System). The approach differs from prior work by the usage of an initial set of ‘seed publications’ for each author; and the expansion of this seed in order to cover the whole oeuvre of each author as represented in a large bibliographic database using an automated process. This automatic process is applied in parallel to each of the authors in the initial set. From these different instantiations of individual oeuvres an ensemble of authors and their publications is built (White 2001). We compare five different approaches to create ‘seed publications’ and three approaches to ‘expand’ the seed. Last but not least, we assess and validate the proposed methodology against a ‘gold standard’ dataset of Dutch authors and their publications that was compiled by the Centre for Science and Technology Studies (CWTS) and verified by the authors themselves.

The proposed methodology is able to account for different kinds of author ambiguity such as different ways of spelling a name, different ways to store a name (initials, first and last name, etc.) in different database systems, and misspellings. Homonyms, i.e., different authors with the same name, can be partially resolved using additional information about authors such as address and institution information. Yet, two authors might work at the same institution and on similar topics and be merged. Note that combining information sources does not automatically lower ambiguity. Each new source adds not only the impurity of the data which requires cleaning but also the challenge of matching the new information to the previous ones. There is much human error that is hard or impossible to detect: Mail addresses can be wrongly noted or allocated, even publications verified by the authors themselves can be wrong. While we aim for a scalable methodology of automatic oeuvre detection we include some manual cleaning to counter these ambiguities both on the initial datasets and on the created mappings between them.

The rest of the paper is organized as follows. First, a description of the different datasets is given, followed by an overview of the ‘seed creation’ approach applied. Second, the ‘expansion of the seed’ and the results and issues of performance are presented. Finally, we discuss the key results of the proposed methodology, draw conclusions, and discuss planned work.

## Data

The datasets used in this study are under active development at DANS (Data Archiving and Networked Services), an institute of the Royal Netherlands Academy of Arts and Sciences (KNAW) and the Centre for Science and Technology Studies (CWTS). DANS promotes sustained access to digital research data and also provides access, via NARCIS.nl, to thousands of scientific datasets, e-publications and other research information in the Netherlands. In addition, the institute provides training and advice, and performs research into sustained access to digital information. CWTS is a centre of excellence in quantitative studies of science. It has conducted numerous bibliometric studies both for research and for evaluation, and compiled extensive data about Dutch researchers.^3^

### NARCIS/NOD database: The Dutch full professor seed

DANS hosts the NARCIS Dutch research information system (Baars et al. 2008) a web portal for a set of databases. One of them is the so-called NOD (Dutch research database) which contains information about forty thousand plus personnel employed at Dutch Research Institutions (universities and other academic institutions). The person database contains metadata such as names, e-mail addresses, but also—for some scholars among them—the Dutch Digital Author Identifier (DAI). Introduced in 2008 in the Netherlands, the DAI assigns a unique identifier to every employee of a Dutch university, university of applied sciences (HBO-*Hoger beroepsonderwijs*) or research institute. Since 2006, NARCIS also harvests publications from Dutch scientific repositories. These are matched to the scholars on their DAI. A complete dump of the NARCIS database was made on April 3, 2012 and is used in this paper (Reijnhoudt et al. 2012). Specifically, we will use the set of 8,378 *hoogleraren*, or *full professors,* and their 1,05,128 papers to exemplify the proposed methodology. For 75 % of the full professors the DAI is recorded in the NOD.

### CWTS Web of Science database: Web of Science publication data

The in-house CWTS version of the Thomson Reuters Web of Science (WoS) consists of nearly 35 million scientific publications and hundreds of millions of citations, from 1980 up to 2012, covering all fields of science. It comprises the Science Citation Index Expanded (SCIE) as well as different enhancements made during the scientific and commercial activities of CWTS over more than 20 years. Enhancements include among others: the standardization of different fields, namely addresses, journal names, references and citation matching, and a new disciplinary classification at the paper level (Waltman and van Eck 2012). The methodology proposed here uses the standardized address information and this new classification.

### CWTS SCOPUS database: Scopus author identifier

Scopus is one of the largest abstract and citation databases of peer-reviewed literature. The database contains 47 million records, 70 % with abstracts from more than 19,500 titles from 5,000 publishers worldwide covering the years 1996–2012 (http://www.info.sciverse.com/ scopus/about). Of particular interest for this study is the newly introduced ‘Scopus Author Identifier’ that is based on an assignment of documents to authors determined by their similarity in affiliation, publication history, subject, and co-authors (Scopus 2009). It has been discussed that articles assigned to a particular Scopus Author Identifier tend to be articles of the author represented by that identifier, but the set of articles might be incomplete, or articles by the same author might be assigned to multiple identifiers (Moed et al. 2012).

### CWTS gold standard dataset: verified individual publication oeuvres

Frequently CWTS’ studies that begin at the individual author level (e.g. ‘bottom–up’ approaches) require a manual verification process in which the individual researchers check and verify their own lists of publications—for more details on this verification process see (van Leeuwen 2007). This verification process has been applied to different sets of researchers in the Netherlands on publications from 2001 to 2010. By manual matching the NARCIS professors on initials, last names and organisations to this dataset of verified author-publication oeuvres we retrieve a ‘gold standard’ dataset. This ‘gold standard’ dataset consists of 1,400 full professors captured in NARCIS and is further used to evaluate different author disambiguation methods. The use of a gold standard set is a common approach in bibliometric and information retrieval research (Costas and Bordons 2008; Sladek et al. 2006).

## Methodology

**Fig. 1:**
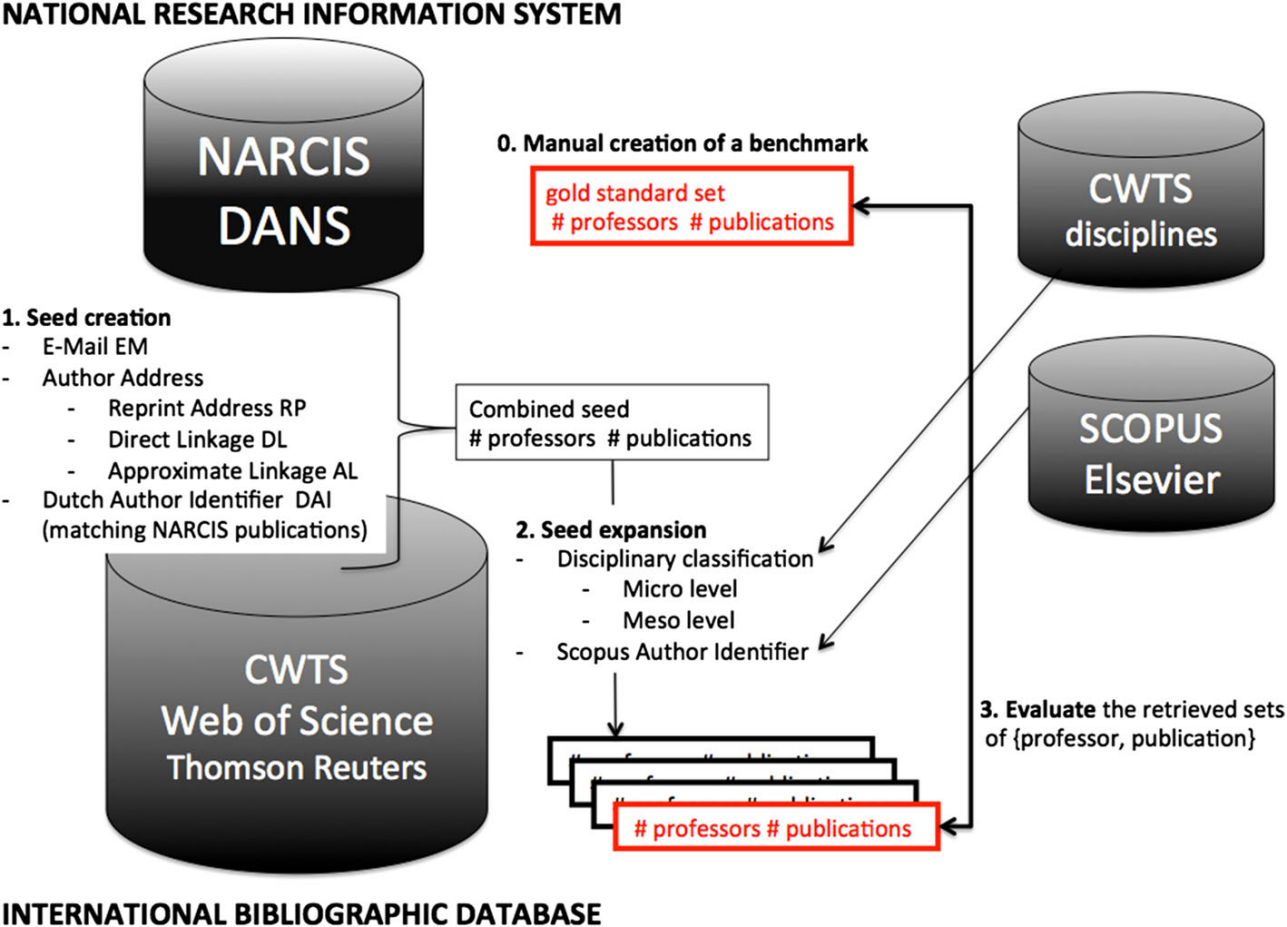
General workflow and relevant data sources

The main objective of this study is to develop and validate a general methodology, called ‘seed + expand’, for automatic oeuvre detection at the individual author level. Given a set of author names, we are interested to detect their publications, as many (high recall) and as correctly (high precision) as possible combining data from different databases. To exemplify the methodology, we use the set of all 8,378 full professors included in the NARCIS database. Figure 1 shows an overview of the workflow comprising:

1. *Seed creation*: Starting with an initial list of 8,378 full professors, we collect information on their name, affiliation, and e-mail addresses from the NOD. Next, we identify ‘seed publications’ in the WoS for each author using five different approaches.
2. *Seed expansion*: Retrieval of additional papers for seed authors based on characteristics of the papers. Three different approaches are compared.
3. *Evaluation*: Results of the different seed expansion approaches are validated using standard measures for precision and recall and the CWTS ‘gold standard’ dataset of 1,400 authors for which verified publications in the period 2001–2010 are available.

All three parts are detailed subsequently.

## Seed creation

The first step of the methodology consists of the creation of a reliable ‘seed’ of publications for the 8,378 target professors. An element of this set consists of a triplet of elements (publication identifier, author name identifier and author position in the paper). The identifiers come from different databases; and the author position indicates if the scholar is first, second, or *n*th author for that publication. The accuracy of the seed is very important as the precision and recall of the final oeuvre detection will be significantly higher if the precision of the seed is high. It is important to bear in mind that during the expansion phase of the methodology it will not be possible to add papers for those professors that are not already in the seed. So, for this phase in the methodology it is not the recall *on papers* but the recall *on authors* that matters. Five different approaches of creating ‘accurate’ seeds are explored here:

- E-mail seed: A seed based on the matching of the e-mail of the professor with the publications in Web of Science.
- Three author-address approaches: These seeds are based on different combinations of the name of the professors and the affiliation(s) of that professor matched with the Web of Science.
- DAI seed: This approach builds upon the publications in NARCIS that have been attached to the professors through the Dutch Author Identifier.

### E-mail seed (EM)

In the NOD system, e-mail addresses are attached to scholars directly or via their affiliations. Hereby, e-mail addresses of the target professors are simply matched against e-mail addresses of authors found in the papers in Web of Science. This approach produced a seed for 4,786 different authors (57 % of the professors from our list) with at least one paper found in WoS, see also Table 1. As e-mail addresses are uniquely attached to one scholar^4^ and are seldom transferred to other scholars,^5^ this approach is assumed to be highly accurate.

**Table 1:** Result sets obtained by different seeds

Seed method	CWTS publications	NARCIS full professors	Full professors unique to this seed
EM	40,826	4,786	790
RP	81,079	5,819	149
DL	79,515	5,749	158
AL	28,837	5,018	76
DAI	30,322	2,742	162
Total unique in combined seed	174,568	6,989	

### Three author-address approaches

Three approaches combine author names and affiliation data in the NOD system and match them to WoS affiliation data to retrieve relevant publications. This Author-Address approach was only feasible thanks to standardization of WoS affiliations and addresses by CWTS. However, some parts of this task also required manual handling and checking. As a result of this, 92 % of the papers with a Dutch organization in the WoS have a matched counterpart in the NOD organizations. These are the only 652,978 papers that can be considered for the Author-Address approach seeds as described in the following paragraphs.

#### Reprint author (RP)

In scientific publications, the reprint address refers to the address of the corresponding author in charge of managing requests that a publication may generate. In the WoS database this reprint address appears directly linked to the author, thus offering a direct and trusted linkage between an author and an organization that can be directly extracted from the publication. Thus, the creation of this seed consists of the matching of the name of the professor and his/her affiliation as is recorded in the NOD with the reprint author name and the reprint affiliation.

#### Direct linkage author-addresses (DL)

**Fig. 2:**
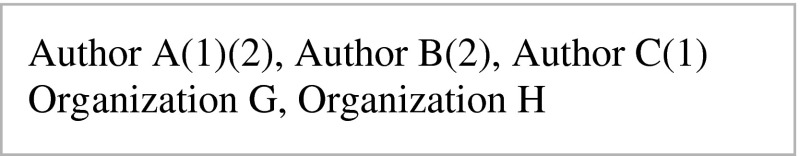
Example of direct author organization linkage

69 % of publications WoS include data on the linkage between the authors and their organizations as they appear in the original publications. For instance, if the original publication featured three authors and two organizations this linkage of authors and organizations is indicated as follows (Fig. 2):

Indicating that Author A is linked to Organization G and H; Author B is linked to Organization H, and Author C is linked to Organization G. As in the RP-based approach, the names and the affiliations of the professors are matched with the author-affiliation linkages of the publications, detecting those publications that, based on this author-affiliation linkage, could belong to the target professors.

#### Approximate linkage author-addresses (AL)

The other 31 % of publications did not have a direct linkage recorded in the database. Thus, authors and affiliations of the publications were recorded, but there was no way to tell which author is affiliated with what organization. This approach detects as seed publications of the target professor all publications that contain the same name and affiliation as the target professor. Therefore, the AL approach has the potential problem of wrongly attributing a publication to a target professor if the name of the author and the institute both appear on a paper. For instance, referring back to Fig. 2, if a homonym of ‘Author B’ (i.e., another scholar with the same name) appears in a paper where ‘Organization H’ also appears, the real ‘Author B’ might get this paper wrongly attributed to him/her.

### DAI seed (DAI)

This seed creation approach starts with publications that are in the NARCIS database attributed by means of the DAI to the target professors. NARCIS retrieves its publications from Dutch open repositories. Consequently, NARCIS does not retrieve the complete oeuvres of these authors. The DAI is not always part of the metadata of the publication, so the NARCIS-publication set is by no means complete. About 70 % of the publications have at least one DAI attached to an author. To complicate matters these publications are not necessarily all Web of Science publications (there may be books, theses, or journal articles not covered in the WoS). For this reason, it was necessary to perform a matching process between the bibliographic records for the professors with a DAI extracted from NARCIS and the WoS database. The NARCIS publications were matched with the WoS publications on journal, year, title, and first page. This way, we were able to create a new seed, based on the publications covered in the WoS that were also in the NARCIS database for the target professors. This seed is also expected to be very accurate in precision, though not in recall.

### Combining the seeds

Table 1 shows the resulting numbers of publications and professors created by the five different seed creation approaches. Publications are counted once per seed, even if they appear several times for different professors. The last column shows the number of professors that were found exclusively by this particular seed method. So, if the AL approach would not be used, the number of professors found would drop only by 76, whereas not using the EM approach would result in a drop of 790 professors. At the end, all seed results are combined (added) and cleaned for duplicates leaving us with 6,989 unique professors and corresponding 1,74,568 publications.

To further improve the quality of the seed, we remove both *multiple assignments* and *common names*. One specific feature of the CWTS database is that it records for every author name—publication pair the author position number. When more than one professor is matched to the same paper and the same author position number we call this a case of *multiple assignment*. Clearly this is wrong, as only one professor should be matched to one author. In order to keep the seed as precise as possible and thus sacrificing some recall for precision, all these records have been removed (see Table 2, ‘remove multiple assignments’). Also, authors with very *common names* could be problematic in the seeds based on author-affiliation combinations (i.e. it is not impossible that within the same institution there are two different professors with the same author name). In order to minimize this problem, the names that belong to the top 5 % most common author names in the Netherlands (first initial-last name pairs) from the former two seed-approaches (RP and DL) and the top 10 % from the least precise approach (AL) have been removed to keep the level of ‘noise’ (i.e., false positives) in the seed to a minimum. (See Table 2, column ‘remove common names’).

**Table 2:** Pruning the seeds to increase precision

Seed method	Number of found professors	Remove multiple assignments	Remove common names
EM	4,786	4,786	4,786
RP	5,819	5,696	4,648
DL	5,749	5,629	4,675
AL	5,018	4,864	3,147
DAI	2,742	2,742	2,740
Total unique in combined seed	6,989	6,947	6,753

The final resulting seed comprises 6,753 professors (80 % of initial set of 8,378) with 1,57,343 unique papers.

## Number of professors without a seed

As previously stated, the approach depends significantly on the ability of detecting a minimum seed of at least one paper for most of the target professors. As a result, the seed expansion will only add papers for those authors that have already an initial seed of publications. Thus, having at least one paper as seed for as many professors as possible is paramount in the process. We have detected that a total of 1,625 (20 %) professors do not get any seed paper. Zooming into these missing professors might offer some insight on the reasons and cases when finding an initial seed may be problematic.

**Fig. 3:**
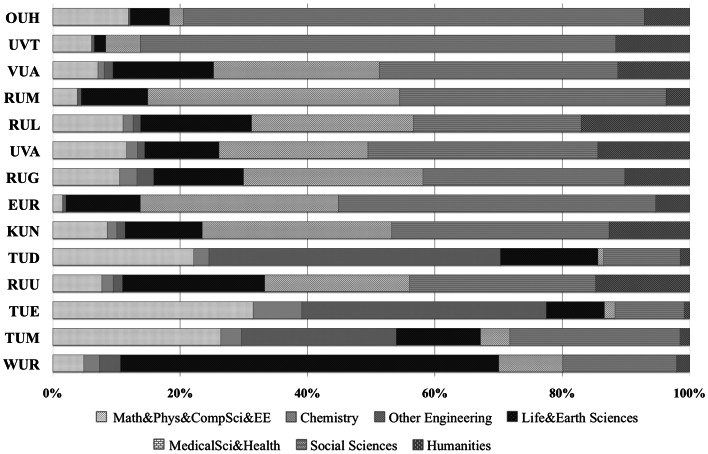
Dutch University profiles, from (2012) NARCIS: Network of Experts and Knowledge Organizations in the Netherlands

Table 3 shows the number of professors per university and the percentage that is missed for lacking a proper seed of publications. From an institutional point of view, all of the major 14 universities in the Netherlands have at least 75 % of their target professors present, except the Open University (50 %) and Tilburg University (32 %). These two universities have an important Social Sciences and Humanities focus, as can be seen in Fig. 3. Both Humanities and Social Sciences are two areas that are not well represented by the WoS, thus explaining their higher presence of professors without a seed.

**Table 3:** Missing professors per university with more than 100 professors

Abbr.	University	No. profs	% Missed	% Found EM
OUH	Open Universiteit - OUNL	124	50	34
UVT	Tilburg University	452	32	26
VUA	VU University Amsterdam	889	24	47
RUM	Maastricht University	552	24	40
RUL	Leiden University	802	21	62
UVA	University of Amsterdam	961	19	60
RUG	University of Groningen	938	18	62
EUR	Erasmus University Rotterdam	668	18	67
KUN	Radboud University Nijmegen	689	17	62
TUD	Delft University of Technology	621	17	67
RUU	Utrecht University	930	16	55
TUE	Technische Universiteit Eindhoven	369	11	73
TUM	University of Twente	358	11	68
WUR	Wageningen University & Research Centre	333	3	84

The EM seed, based on e-mail addresses, yielded the most unique identified professors of all the seeds, as can be seen in Table 1. The percentage of found professors on e-mail per university is shown in the last column of Table 3. This is not equal for all universities, notably the Wageningen University (WUR), which only misses 3 % of its professors in our seed, yielded 84 % of the professors found on e-mail information. As a partial explanation for this difference we can mention the different collaboration patterns among fields (Larivière et al. 2006; Sonnenwald 2005; Costas and van Bochove 2012). When the number of co-authors in a paper is high, the probability is low that the one with the e-mail is the author in our seed. The WUR is a university focused on the Life Sciences and particularly on agricultural topics. It is possible that the lower collaboration level in these topics compared to other more collaborative fields (e.g. Physics) can be among the explanations for this higher presence of the e-mail addresses of WUR scholars. In those cases where only one e-mail is recorded by the journal, the fact that there are fewer co-authors (as compared to other fields) increases the possibilities that the e-mail recorded in the Web of Science is from a scholar from WUR university.

Overall, the percentage of professors with a known e-mail address is over 94 % for these fourteen universities, and the percentage of their professors found on e-mail address is about 60 %. This supports the idea of e-mail data as a powerful source of information for the identification of individual scholars and its possibilities as an important parameter for author name disambiguation.

To explore another perspective on the professors we missed, we group the persons by type of affiliation. Since some professors have multiple affiliations, the sum of the third column of Table 4 is higher than the total amount of professors in the initial NOD-set.

**Table 4:** Percentage missed professors by affiliation in the first three seeds

Job title	Abbr.	No. profs	% Missed	Found on EM
Visiting professor	GHL	46	61	22
Rector	RMA	11	55	36
Honorary professor (without salary)	OHL	119	41	34
Honorary professor	HHL	18	39	44
Extraordinary professor	BHL	1,380	28	46
Dean	DCN	78	26	44
Part-time professor	PTH	300	25	41
Professor emeritus	EMT	38	21	37
Professor	HGL	4,596	18	60
Associate professor	UHD	2,465	14	61
Management	DIR	612	14	66
Researcher	OND	557	11	71
Contact person organisation	CPO	58	9	74
University professor	UHL	29	7	68
Project leader	PRL	162	2	85

The highest missed percentages are on Visiting Professors and Rectors. The Visiting Professors also have a very low recall on e-mail, this could be due to the fact that while they visit the Netherlands, they are known by a Dutch email address in the NARCIS-system, used in this seed, while their work will be known by their original foreign e-mail address. Of the Rectors who also have a professorship only 5 out of 11 are present. This situation could be caused by the fact that these are senior scholars who spend much of their current professional time on service activities. Past scholarly publications are likely outside the time period included in this study, or as older publications their matching with the original authors is more difficult as the author-affiliation linkages are more robust for the most recent period. Two Rectors have dedicated rector e-mail addresses, which they will not have used on any papers, three are employed by smaller universities, specialized in theology or humanities, which are not well represented by WoS.

This exploration shows that creating a seed based on e-mail addresses, though it yields the best overall result, might not be the best method for some positions as well as some universities and subject fields. This sheds a light on the complexity of the disambiguation as well as on the value of combining multiple seed creating methods.

## Seed expansion

In this second phase, we use the 6,753 author profiles and associated papers in the seed to identify additional publications by these authors in the WoS database. Three approaches have been explored and are detailed subsequently.

### Two CWTS paper-based classifications (meso and micro)

These two approaches use a new paper-based classification that has been developed at CWTS (Waltman and van Eck 2012) based on the citation relationships of individual publications. It has been applied to publications between 2001 and 2010, excluding the Arts and Humanities. The hierarchical classification has three levels, with a medium-level classification that comprises 672 ‘meso-disciplines’, and a lower-level classification that includes more than 20,000 different ‘micro-disciplines.’ We assume that within small disciplinary clusters (meso and micro) there is a rather low probability that two professors share the same name. Hence, we assign all papers within a meso/micro-discipline that have the same author name to one professor. Incorrect assignments might occur when two persons with the same name work in the same subfield.

Performing assignments at the meso-discipline level results in an increase of 34 % to 2,11,202 unique papers, the micro-disciplines yield a subset thereof with 1,94,257 unique papers, an increase of 23 %.

### Scopus author identifier approach

**Fig. 4:**
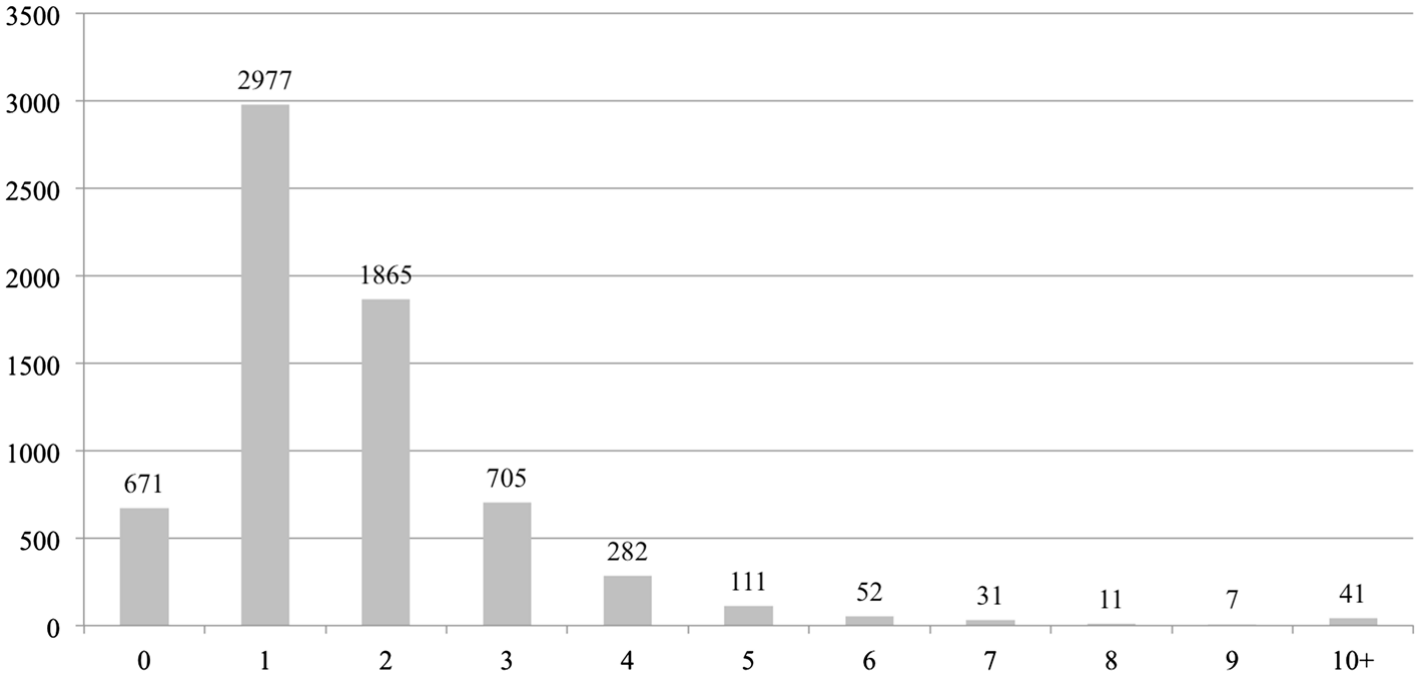
Number of authors (*y*-axis) from the seed with the number of matched Scopus author identifiers (*x*-axis). The 41 authors with ten or more were ultimately discarded

A third approach to expand the publications of professors is to use one of the already existing author identifiers. We choose the Scopus Author Identifier because it has been introduced for all authors in the Scopus database. Here, the 1,57,343 WoS publications from our initial seed were matched to Scopus publications and their 6,753 authors were matched with Scopus authors to derive their Scopus author identifier. As shown in Fig. 4, 671 WoS seed authors had no Scopus author identifier; 2,977 authors had exactly one Scopus author identifier; and all others had more than one. This indicates that Scopus author identifiers err on the side of recall rather than on precision. All Scopus author identifiers were used to retrieve additional Scopus publications that were traced back to WoS publications via bibliographic matching on journal, title, etc. The resulting set has 2,66,105 unique papers, an increase by 69 %—the largest number of publications of the two expansion approaches.

## Evaluation

By using multiple sources that all contain some erroneous data, our method might multiply those errors. Matching specific records introduces even more possible errors. So to evaluate the three seed expansion approaches, their result sets are compared to the CWTS gold standard dataset introduced in the Data section. The expansion of the seed by the different approaches has been performed on the whole WoS (from 1980 to 2011). But to evaluate the approaches we restrain the result of the expansion to publications published between 2001 and 2010, the same time period as the gold standard set. Exactly 1,400 of the 6,753 authors (21 %) are in the gold standard dataset—only 63 professors are not accounted for. These 1,400 authors and their 57,775 associated papers will be used to measure precision and recall achieved by the different approaches.

**Fig. 5:**
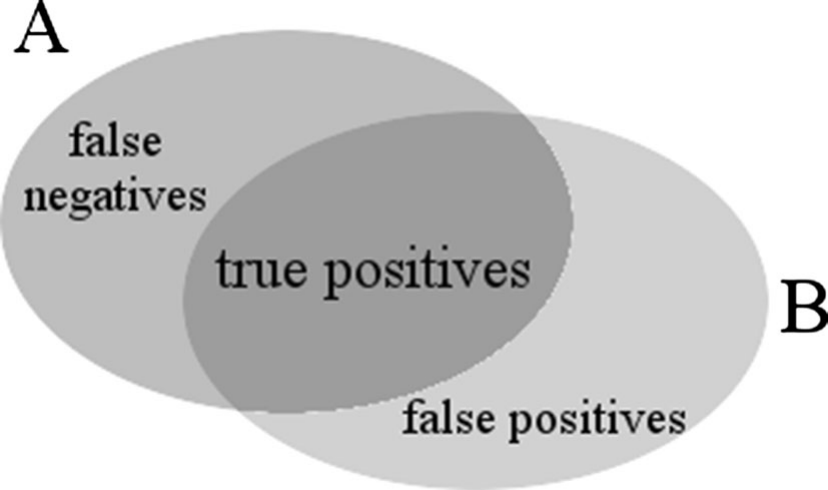
Gold standard set (*a*) versus the result of the expansion (*b*)

Precision and recall are widely used to measure how well an information retrieval process performs. Precision is defined as the retrieved relevant records (true positives) divided by all retrieved records (both true and false positives). Recall on the other hand is the number of retrieved relevant records divided by the number of records that should have been retrieved (the true positives and the false negatives). Thus, we can score the performance of our different approaches according to these two parameters, see Table 5 (Fig 5).

**Table 5:** Performance of the three expansion approaches—individually and combined

	Scopus identifier	Meso	Micro	ScopusI and Meso	ScopusI and Micro
True pos. (A∩B)	55,405	55,459	55,394	55,509	55,460
False pos. (¬A∩B)	8,055	10,430	7,212	13,200	10,260
False neg. (A∩¬B)	2,370	2,316	2,381	2,260	2,315
Precision	87.3	84.2	88.5	80.8	84.4
Recall	95.9	96.0	95.9	96.1	96.0
F_1_	45.7	44.9	46.0	43.9	44.9

The three first columns in Table 5 present the different expansion approaches individually. In general, the values for recall are equally high for the three approaches. Regarding the precision there is a slight difference. As expected, the micro-discipline set has a higher precision and lower recall than the meso-disciplines approach. The Scopus author identifier approach, with 63,460 professor-paper combinations and a precision of 87.3, ends up in between. The F-score shows the same order, since there is hardly any difference in recall.

The last two columns show the combination of two approaches: *Scopus author id plus meso*-*disciplines* and *Scopus author id plus micro*-*disciplines*. As can be expected, the recall increases, whereas the precision declines. The increase in the recall is rather small, and the number of false negatives is high. This indicates that both approaches miss roughly the same papers. Apparently there are some publications in the oeuvres of some researchers that are hard to find using the kind of approaches presented in this paper.

## Conclusions

Of the 8,378 professors in our target list we identified at least one publication for 6,753 (80 %) professors, which gave us a seed into their oeuvre (as far as covered by the WoS). Combining all publications of all professors we started with a set of 6,753 authors and their 1,57,343 unique publications. After the expansion of the individual publication seeds with the Scopus author id approach and the micro-disciplines approach we find the same recall on the gold standard dataset, and a comparable precision, as shown in Table 5. Both approaches introduce roughly the same percentage of wrongly attributed papers. The Scopus author id approach finds more unique papers (2,66,105 vs. 1,94,257). This can be attributed to the restrictions on the disciplines classification on period (2001–2011) and disciplines, e.g., the Arts and Humanities WoS publications are not included (Waltman and van Eck 2012).

The gold standard dataset used for evaluation covers 1,400 (or 21 %) of the 6,753 professors and we assume the precision and recall results can be extrapolated to the entire data collection. It is important to remark that the results of precision are slightly conservative due to the fact that for some authors some publications were still missing even in their verified set of publications. This happens particularly with authors with high numbers of publications, in fact Smalheiser and Torvik (2009) indicated that this happens when authors have more than 300 publications. In other words, although the precision of our gold standard set is 100 % (basically we can assume that all are correct publications, as verified by their authors) it seems that the recall of the golden standard set is not necessary 100 %. Thus, the values of ‘wrong’ publications obtained through our methodologies might not be as high in reality, and thus we can consider this measure to be the upper bound of false positives that we could expect for the whole analysis, because true values will likely be smaller. The methodology developed in this paper will be further applied in an impact study of the set of Dutch full professors, retrieving citations to all their publications. We would like to point out that our methodology, relying on domain specific scholarly communication, is sensitive towards the disciplinary composition of the author set, e.g., authors that publish mostly books are underrepresented. This will be explored in further analysis.

Note that the success of cross-database retrieval and author disambiguation heavily depends on access policies of the hosting institutions, and the quality of the databases involved. Even if access is given, extensive institutional collaboration is required to interlink and harmonize databases. Initiatives such as ORCID (Foley and Kochalk 2010) with the idea of a central registry of unique identifiers for individual researchers or bottom-up networked approaches such as the VIVO international researcher network (Börner et al. 2012) that assigns unique VIVO identifiers to each scholar, aim to provide processes and data structures to assign and keep track of unique scholars and their continuously evolving oeuvres. The data collected by ORCID and VIVO can be used as additional ‘gold standards’ in future evaluation studies. For example, the methodology presented here can be applied to retrieve publications for scholars with a valid ORCID and VIVO from the existing commercial and public data sources. Ultimately, unique author identifiers are required for the comprehensive analysis of science, e.g., using altmetrics (Wouters and Costas 2012), and also for models of science (Scharnhorst et al. 2012) using data from multiple databases.

## Footnotes

1 The complexity of name disambiguation seems to rise if one goes back in history, which also has to do with a cultural change in claiming authorship. As stated in Wikipedia (http://en.wikipedia.org/wiki/Medieval_literature) in the medieval literature one finds a tendency to anonymity or referring to an auctor (http://en.wikipedia.org/wiki/Auctor). Not to talk about different forms of names in Latin, Greek or other languages and their changes over time.

2 The idea of combining different data sources with the objective of collecting data at the individual level is not completely new (see for example D’Angelo et al. 2011) and has shown already interesting results.

3 The data compiled at CWTS about Dutch scholars are used for the research purposes in this study only and with consent of the owner.

4 Exceptions exist with addresses like info@ or dep@.

5 We expect sometimes an e-mail is transferred to another researcher with the same (or very similar) name in the same organization when the previous e-mail holder has left the organization.
